# Supplementary material for: Crawling and Gliding: A Computational Model for Shape-Driven Cell Migration
Source: PLoS Comput Biol. 2015 Oct 21;11(10):e1004280. doi: 10.1371/journal.pcbi.1004280 (PMC4619082; doi:10.1371/journal.pcbi.1004280)
Supplement: S1 Code — (ZIP) [file pcbi.1004280.s012.zip › release/tst/doc/html/classDir.html]

Tissue Simulation Toolkit: Dir Class Reference


Tissue Simulation Toolkit
 0.1.4.1


- Main Page
- Namespaces
- Classes
- Files

- Class List
- Class Hierarchy
- Class Members

Public Member Functions |
Public Attributes |
Friends |
List of all members

Dir Class Reference

`#include <ca.h>`

|  |  |
| --- | --- |
| Public Member Functions | |
|  | Dir () |
|  | |

|  |  |
| --- | --- |
| Public Attributes | |
| double | aa1 |
|  | |
| double | aa2 |
|  | |
| double | bb1 |
|  | |
| double | bb2 |
|  | |
| double | lb1 |
|  | |
| double | lb2 |
|  | |

|  |  |
| --- | --- |
| Friends | |
| class | CellularPotts |
|  | |

## Constructor & Destructor Documentation

|  |  |  |  |  |
| --- | --- | --- | --- | --- |
| Dir::Dir | ( |  | ) |  |

inline

References aa1, aa2, bb1, bb2, lb1, and lb2.

## Friends And Related Function Documentation

friend class CellularPotts

friend

## Member Data Documentation

double Dir::aa1

Referenced by Dir(), CellularPotts::FindCellDirections(), and CellularPotts::ShowDirections().

double Dir::aa2

Referenced by Dir(), and CellularPotts::FindCellDirections().

double Dir::bb1

Referenced by Dir(), CellularPotts::FindCellDirections(), and CellularPotts::ShowDirections().

double Dir::bb2

Referenced by Dir(), and CellularPotts::FindCellDirections().

double Dir::lb1

Referenced by Dir(), and CellularPotts::FindCellDirections().

double Dir::lb2

Referenced by Dir(), and CellularPotts::FindCellDirections().

---

The documentation for this class was generated from the following file:

- ca.h


---

Generated on Thu Aug 14 2014 22:04:01 for Tissue Simulation Toolkit by  

 1.8.6
